# In_2_O_3_ Nanotower Hydrogen Gas Sensors Based on Both Schottky Junction and Thermoelectronic Emission

**DOI:** 10.1186/s11671-015-1002-4

**Published:** 2015-07-15

**Authors:** Zhao Qiang Zheng, Lian Feng Zhu, Bing Wang

**Affiliations:** Institute of Micro-nano Optoelectronic Technology, Shenzhen Key Lab of Micro-nano Photonic Information Technology, College of Electronic Science and Technology, Shenzhen University, Shenzhen, 518060 Guangdong People’s Republic of China; State Key Laboratory of Optoelectronic Materials and Technologies, Nanotechnology Research Center, School of Physics & Engineering, Sun Yat-sen University, Guangzhou, 510275 Guangdong People’s Republic of China; Department of Materials Science and Engineering, Tsinghua University, Beijing, 100084 People’s Republic of China

**Keywords:** Nanotowers, Hydrogen, Gas sensor, Schottky junction, Thermoelectronic emission

## Abstract

Indium oxide (In_2_O_3_) tower-shaped nanostructure gas sensors have been fabricated on Cr comb-shaped interdigitating electrodes with relatively narrower interspace of 1.5 μm using thermal evaporation of the mixed powders of In_2_O_3_ and active carbon. The Schottky contact between the In_2_O_3_ nanotower and the Cr comb-shaped interdigitating electrode forms the Cr/In_2_O_3_ nanotower Schottky diode, and the corresponding temperature-dependent *I*-*V* characteristics have been measured. The diode exhibits a low Schottky barrier height of 0.45 eV and ideality factor of 2.93 at room temperature. The In_2_O_3_ nanotower gas sensors have excellent gas-sensing characteristics to hydrogen concentration ranging from 2 to 1000 ppm at operating temperature of 120–275 °C, such as high response (83 % at 240 °C to 1000 ppm H_2_), good selectivity (response to H_2_, CH_4_, C_2_H_2_, and C_3_H_8_), and small deviation from the ideal value of power exponent β (0.48578 at 240 °C). The sensors show fine long-term stability during exposure to 1000 ppm H_2_ under operating temperature of 240 °C in 30 days. Lots of oxygen vacancies and chemisorbed oxygen ions existing in the In_2_O_3_ nanotowers according to the x-ray photoelectron spectroscopy (XPS) results, the change of Schottky barrier height in the Cr/In_2_O_3_ Schottky junction, and the thermoelectronic emission due to the contact between two In_2_O_3_ nanotowers mainly contribute for the H_2_ sensing mechanism. The growth mechanism of the In_2_O_3_ nanotowers can be described to be the Vapor-Solid (VS) process.

## Background

Chemical sensors based on semiconductor oxide materials have been extensively researched due to their advantageous features, such as high sensitivity, low cost, and simplicity in fabrication [1]. Among them, indium oxide (In_2_O_3_) semiconductive materials have been extensively studied as chemical sensors for a long time due to their advantageous features such as a wide bandgap around 3 eV, a low resistance, and good catalysis [2].

1-D In_2_O_3_ nanostructures show high gas-sensing response due to their high electric conductance, high transparency to visible light, strong interaction with the gas molecules, high surface-to-volume ratio, and large surface activities [3]. However, up to now, few In_2_O_3_ nanostructures exists good sensing properties to hydrogen. Hydrogen is the most attractive and sustainable energy for the future generations due to its high efficiency and renewable properties.

In this contribution, we report that new In_2_O_3_ tower-shaped nanostructures with high surface-to-volume ratio have been fabricated using thermal evaporation of the mixed powders of In_2_O_3_ and active carbon. The synthesized In_2_O_3_ nanotowers distributing on Cr comb-shaped interdigitating electrodes with relatively narrower interspace of 1.5 μm have excellent gas-sensing characteristics to hydrogen concentration ranging from 2 to 1000 ppm at operating temperature of 120–275 °C.

## Methods

The fabrication of In_2_O_3_ nanotower hydrogen sensors includes two parts. One is the preparation of comb-shaped interdigitating electrodes with relatively narrower interspace of 1.5 μm on the silicon substrate, and the corresponding processes are basically the same with our previous works [4, 5].

Another part is the synthesis of In_2_O_3_ nanotowers on the silicon substrate by thermal evaporation of active carbon and In_2_O_3_ powders. The active carbon and In_2_O_3_ powders are mixed in a 1:1 weight ratio as the reaction source and put near the silicon substrate, which are placed inside the little quartz tube that is pulled into a large quartz tube in a horizontal tube electric furnace. After the whole system is evacuated by a vacuum pump for 20 min, the argon gas is guided into the system at 250 sccm and the pressure is kept at 350 Torr. Then, the system is rapidly heated up to 1050 °C at 40 °C/min from the room temperature and kept this temperature for 1 h. Finally, the system is cooled down to the room temperature for several hours by natural cooling. The In_2_O_3_ nanotowers are synthesized. Field emission scanning electron microscopy (FESEM), x-ray diffraction (XRD), and high-resolution transmission electron microscopy (HRTEM) are used to identify the morphology and structure of the products.

**Fig. 1:**
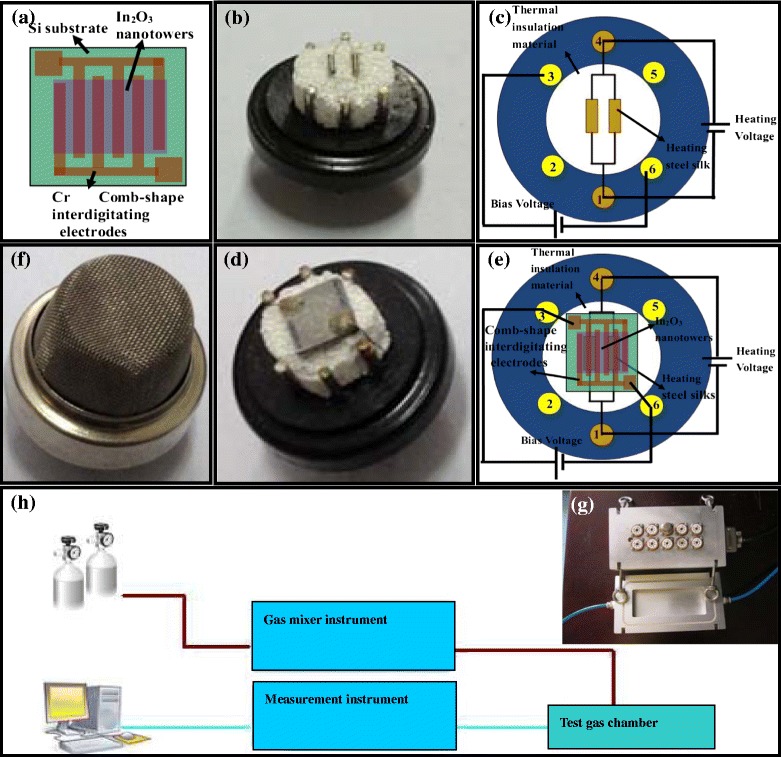
**a** The schematic diagram of the gas sensor chip. **b**, **c** The heater of the gas sensor and the corresponding schematic diagram. **d**, **e** The sensor element and the corresponding schematic diagram. **f** The sensor element packaged with a stainless steel mesh cap. **g** The test gas chamber. **h** The schematic diagram of the gas-sensing characterization system

After that, the In_2_O_3_ nanotowers are scraped down from the silicon substrate and ultrasonically dispersed in ethanol for 30 min to form a suspension. Then, the suspension is taken out by straw and sprinkled on the Cr comb-shaped interdigitating electrodes so as to constitute a gas sensor chip after the ethanol evaporation. The corresponding schematic diagram of the gas sensor chip is shown in Fig. 1a. The Schottky contact between the In_2_O_3_ nanotower and the Cr comb-shaped interdigitating electrode forms the Schottky diode. The corresponding temperature-dependent (25–240 °C) *I*-*V* measurements are performed by using two regulated DC power supplies (RICH/RS1303DQ) and a digital multimeter (VICTOR/VC890D).

The heater of the gas sensor and the corresponding schematic diagram are shown in Fig. 1b, c. The heater steel silks on the thermal insulation material are connected with the metal pins “1” and “4.” Each gas sensor chip is put on the heater steel silks and bonded to another two metal pins so as to constitute a sensor element. The sensor element and the corresponding schematic diagram are shown in Fig. 1d, e. When the heating voltage is applied to metal pins “1” and “4,” the temperature of the heater steel silks will rise up according to Joule’s law so as to make the temperature of gas sensor chip rise up. When the bias voltage is applied to metal pins “3” and “6,” the electrical signal measurement for the gas sensor can be carried out. The sensor temperature at different heating voltages is measured by contacting a thermocouple to the upper side of the gas sensor chip.

Each sensor element is packaged with a stainless steel mesh cap as shown in Fig. 1f. After that, the six metal pins of each sensor element are inserted in the corresponding holes of one measurement unit in the test gas chamber as shown in Fig. 1g. There are 10 measurement units in the test gas chamber, so 10 sensor elements can be tested at the same time. Then, the test gas chamber is sealed. By controlling gas-sensing characterization system (Gyjf Technology Co. Ltd., People’s Republic of China/JFO2E) consisting of gas mixer instrument, measurement instrument, and computer, certain concentration gas or air is passed into the test gas chamber based on a flow-through technique in different circumstances [6], and the corresponding schematic diagram is shown in Fig. 1h. In our case, the concentration of hydrogen gas is ranging from 2 to 1000 ppm. At the same time, bias voltage of 8.9 V is as working voltage, and certain heating voltage ranging from 3 to 5.1 V is as heating voltage which is supplied to test the gas chamber so as to apply to each tested sensor element. The resistance change of the gas sensor can be caught by the measurement instrument and displayed on the computer.

## Results and Discussion

**Fig. 2:**
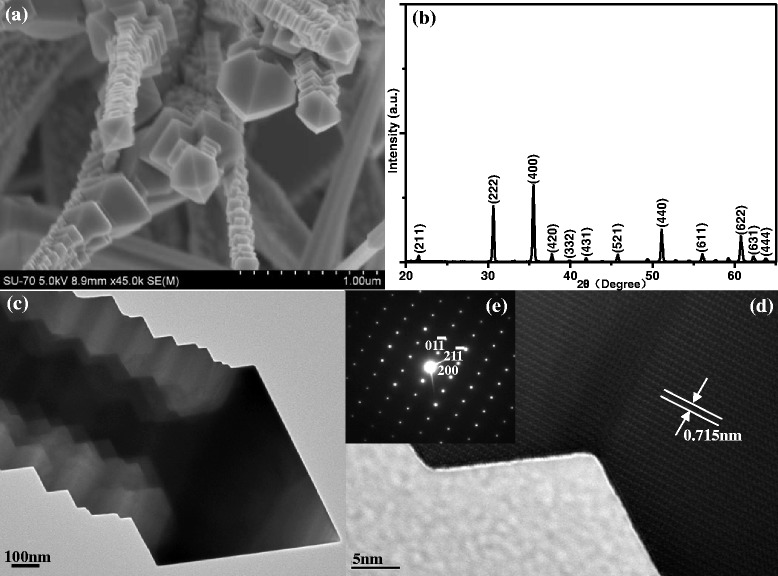
**a** The highly magnified FESEM image and **b** the XRD pattern of the In_2_O_3_ nanotowers [7]. **c** The bright-field image, **d** the HRTEM image, and **e** the SAED pattern of an individual In_2_O_3_ nanotower [7]

The characteristics results of the In_2_O_3_ nanotowers shown in Fig. 2 have been displayed in our previous work which is about the field emission properties of the In_2_O_3_ nanostructures [7]. The highly magnified FESEM image of the synthesized In_2_O_3_ nanotowers is shown in Fig. 2a. The four sides of the nanotower are chucked up with octahedrons one after another so that the nanotower posses high surface-to-volume ratio property. The corresponding XRD pattern of samples in Fig. 2b shows that the fabricated nanostructures are indexed to the cubic In_2_O_3_. According to PDF No. 06-0416, the lattice constants of the cubic In_2_O_3_ are *a* = 10.118 Å, *b* = 10.118 Å, and *c* = 10.118 Å, respectively. Figure 2c is a typical TEM bright-field image of an individual In_2_O_3_ nanotower with an octahedral cap size of 600 nm. The HRTEM image shown in Fig. 2d is recorded at the body section of the In_2_O_3_ nanotower in Fig. 2c. The interplanar spacing of 0.715 nm is corresponding to the (011) crystallographic plane of cubic In_2_O_3_ lattice, and the corresponding selected area electronic diffraction (SAED) pattern in Fig. 2e recorded with an electron beam perpendicular to the surface of the In_2_O_3_ nanotower demonstrates that the In_2_O_3_ nanotower is single crystal and the growth direction is along [200].

**Fig. 3:**
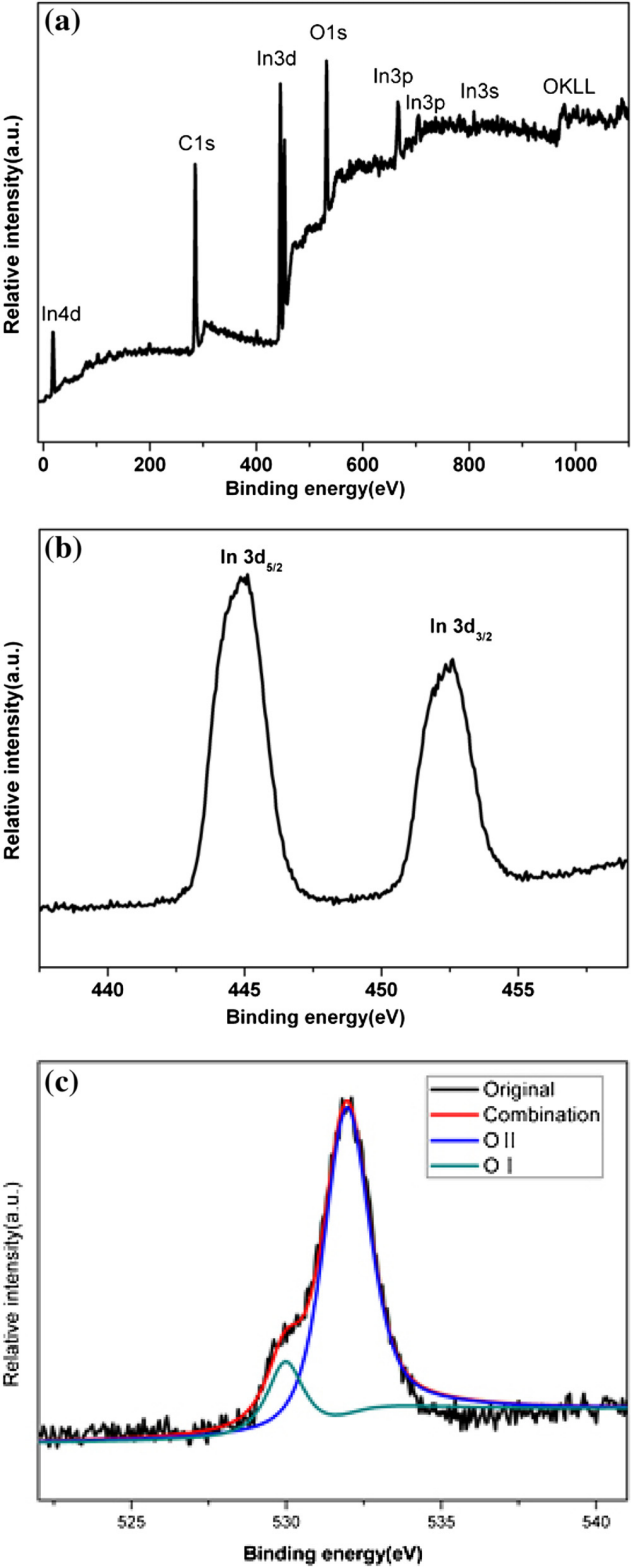
**a** Wide-scan XPS spectrum of the In_2_O_3_ nanotowers. **b** Its spectrum of In 3d. **c** Its spectrum of O1s

The whole synthesis is conducted under the reducing atmosphere. So, the stoichiometric proportion of In and O has been further identified by quantitative x-ray photoelectron spectroscopy (XPS) analysis (in Fig. 3) performed on the product. A wide-scan XPS spectrum shown in Fig. 3a indicated that the product is consisted mainly of In and O. The contaminated carbon is attributed to a small amount of graphite from the reaction source. For its XPS spectrum of In 3d shown in Fig. 3b, because of the spin-orbital splits, In 3d_5/2_ and In 3d_3/2_ have characteristic double peaks located at 444.96 and 453 eV, respectively, corresponding to the binding energy of In^3+^ in In_2_O_3_ [8]. For its XPS spectrum of O1s shown in Fig. 3c, the O1s peaks can be divided into two peaks, centered at 530 eV (OI) and 532 eV (OII), respectively. The OI sub peak is assigned to the lattice oxygen in the In_2_O_3_ nanotower [8–11], and the OII sub peak is usually associated with the chemisorbed oxygen ions in the oxygen vacancy (V_o_) region [8, 10, 11]. The atomic ratio of O:In was 4.4:1, which did not agree with the stoichiometric proportion of O and In presenting in In_2_O_3_. Excrescent O may be adsorption oxygen [12]. As the area ratio of OI/OII is about 1/4.6, so the chemical formula of the nanotowers is In_2_O_1.57_, implying that a large amount of oxygen vacancies are introduced in the product.

**Fig. 4:**
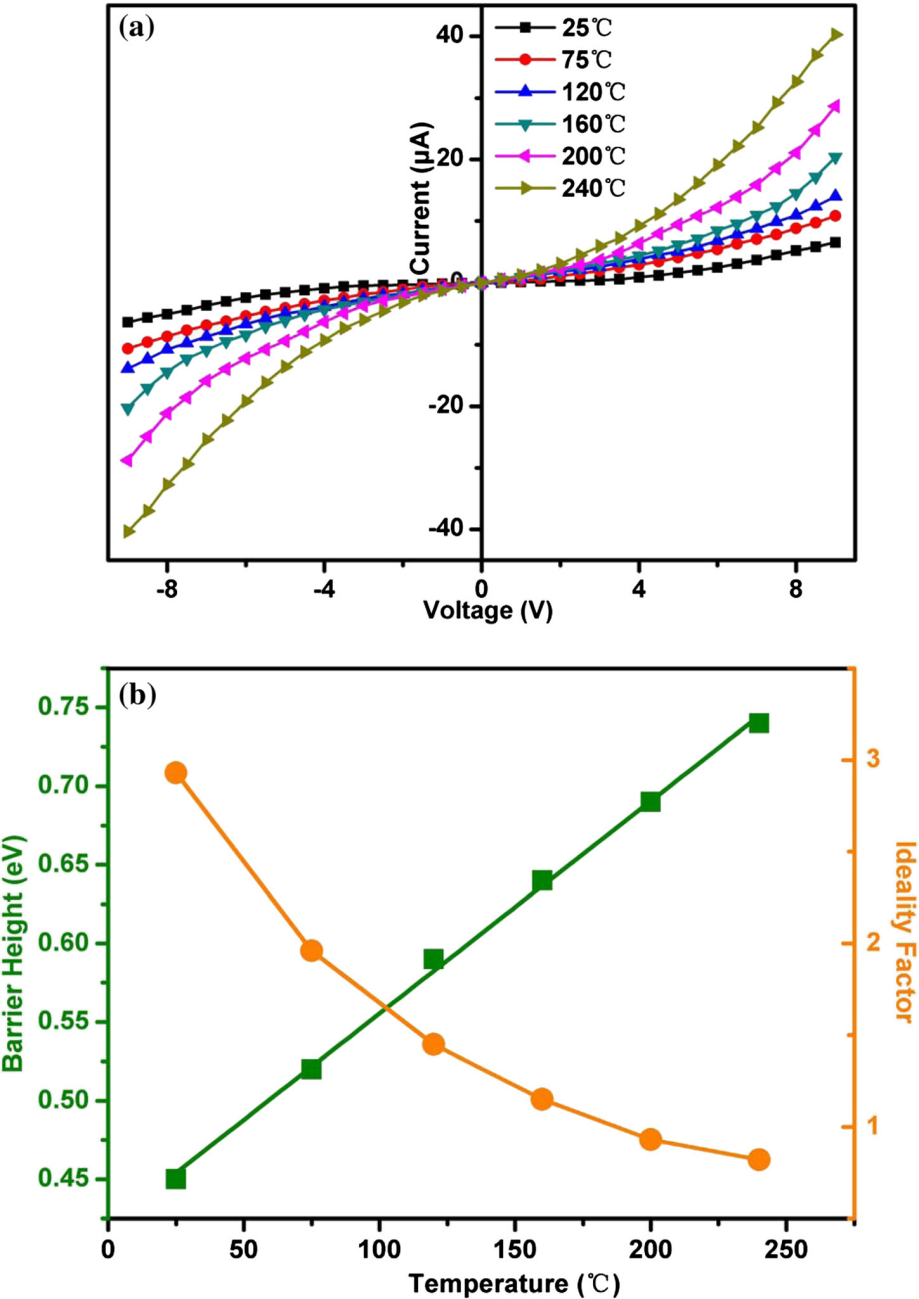
**a** Temperature-dependent *I*-*V* characteristics for representative Cr/In_2_O_3_ nanotower Schottky diode before H_2_ exposure. **b** Variation of barrier height (*φ*_B_) and ideality factor (*n*) with temperature for Cr/In_2_O_3_ nanotower Schottky diode

Temperature-dependent *I*-*V* plots for representative Cr/In_2_O_3_ nanotower Schottky diode before exposure to H_2_ are shown in Fig. 4a. The primary conduction mechanism at Schottky diodes, in general, is due to the flow of majority charge carriers over the barrier by a thermionic process [13]. The electrical characterization of a Schottky diode necessitates the determination of the barrier height and the ideality factor. For an ideal diode, the diode ideality factor should be nearly equal to unity. But in a real situation, it may increase when the effects of series resistance, leakage current, etc. come into play [13]. Depending on the temperature (*T*) and applied voltage (*V*), different transport mechanisms might be simultaneously operative in the Schottky diode and modulate the charge transport. Assuming that the thermionic emission is the most predominant mechanism, the general form of the temperature dependence of the current may be expressed as [13–15]:

1 $$ I=A{A}^{\ast } \exp \left(-\beta {\varphi}_{\mathrm{B}}\right) \exp \left[\frac{\beta \left(V-IR\right)}{n}\right] $$

where, *φ*_B_ is the effective barrier height, *A* is the junction area, A* is the Richardson constant, *n* is the ideality factor, *R* is the series resistance, and *β* = *q*/*kT*. In such a case, the generalized Norde method can be used to evaluate the effective barrier height and diode ideality factor from *I*-*V* measurements [13, 14]. The effective barrier height and ideality factor measured at different temperatures for Cr/In_2_O_3_ nanotower Schottky diode are shown in Fig. 4b. The barrier height can be seen to increase linearly with temperature. On the other hand, the ideality factor for the Schottky diode decreases from 2.93 to 0.82 with an increase in temperature. Deviation of the ideality factor from unity may be due to the existence of high series resistance [14].

**Fig. 5:**
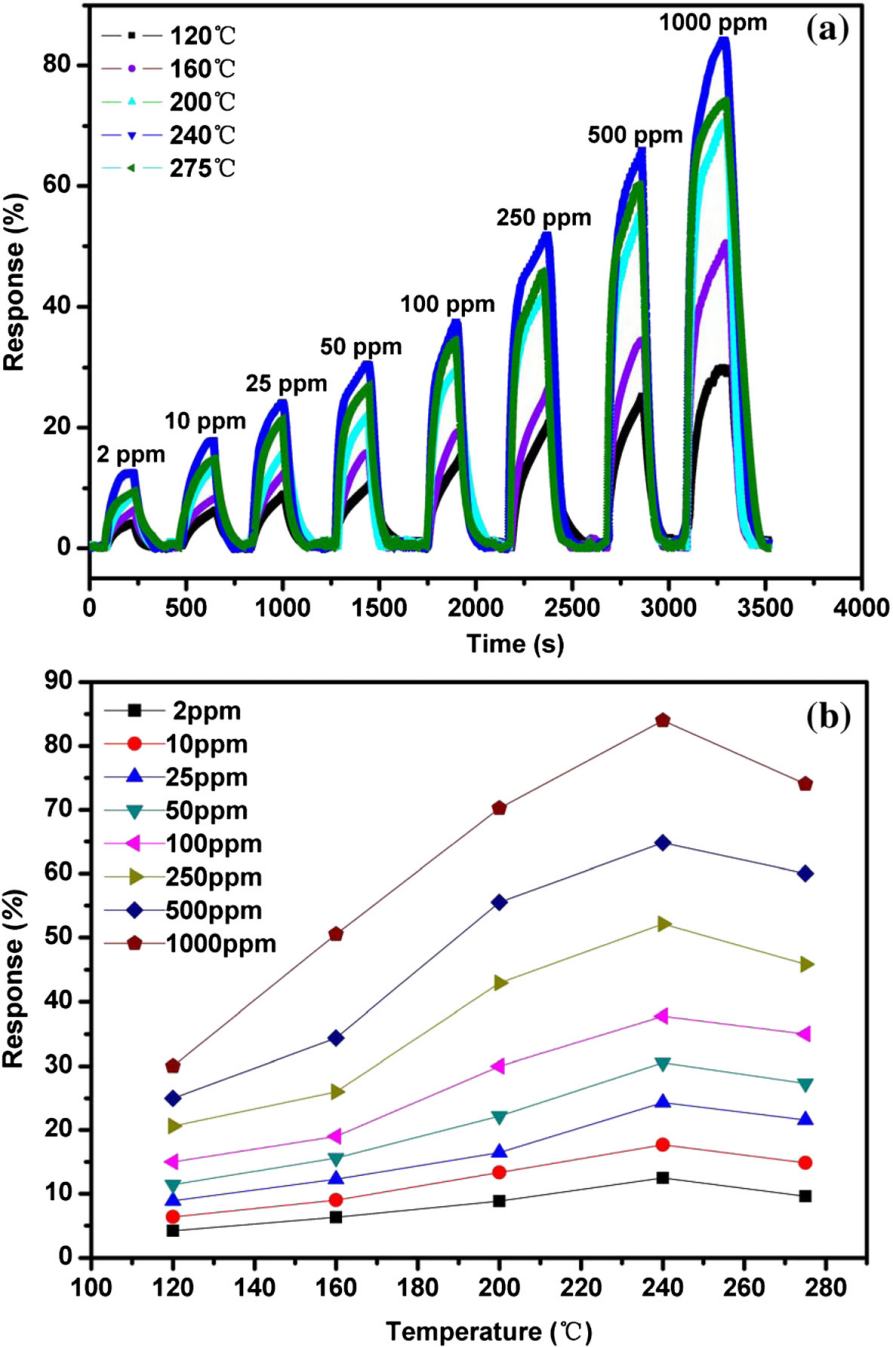
**a** Typical response curves and **b** the response as a function of operating temperature of In_2_O_3_ nanotower gas sensor exposed to H_2_ concentrations ranging from 2 to 1000 ppm and tested at 120–270 °C, respectively

H_2_ sensing characteristics are investigated when the sensor are exposed to different H_2_ concentrations ranging from 2 to 1000 ppm. The different heating voltages of 3, 3.5, 4, 4.5, and 5.1 V correspond to the different substrate temperatures of 120, 160, 200, 240, and 275 °C, respectively. The measurement time is 3500 s in the whole test process. Sensor resistances decrease abruptly upon exposure to H_2_ and recover to initial values after purging by dry air. After several circles between the test gas and dry air, the In_2_O_3_ nanotowers sensor can still recover to the initial states, indicating good reversibility. The responses of the sensor are shown in Fig. 5a. We calculated the response of the sensor using the expression of Response% = (*R*_o_−*R*_g_)/*R*_o_*100 % [16]. Here, *R*_o_ and *R*_g_ are the resistance of the sensor before and after exposure to the tested gas, respectively. Figure 5b shows the response as a function of operating temperatures from 120 to 275 °C for the sensor exposed to different H_2_ concentrations of 2–1000 ppm. In_2_O_3_ nanotower gas sensor reaches the maximum response value of 83 % at an optimal operating temperature of 240 °C.

**Fig. 6:**
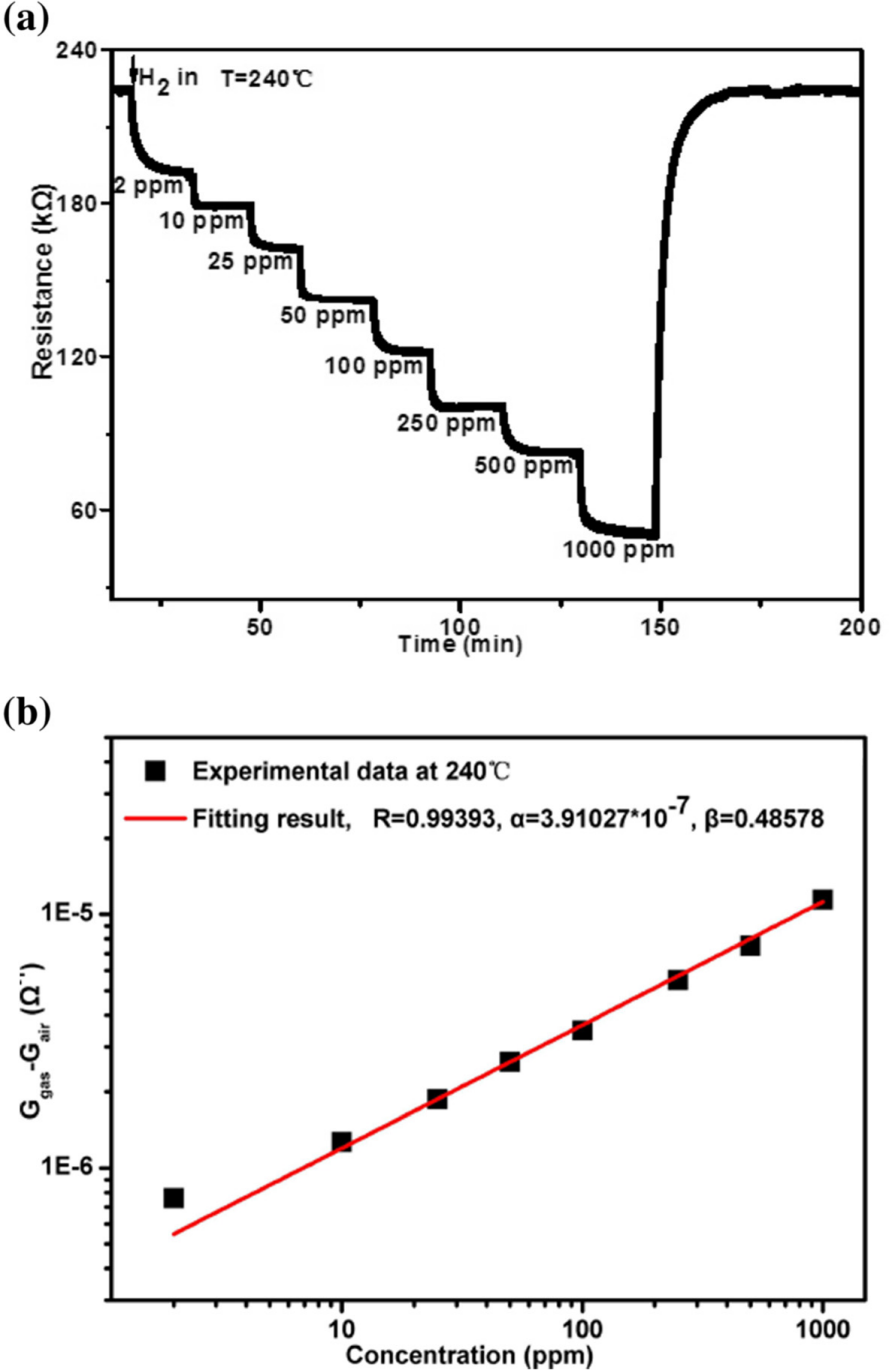
**a** The steady-state resistance responses of the sensor during exposure to increasing H_2_ concentrations (between 2 and 1000 ppm) under an optimal operating temperature of 240 °C. **b** The corresponding steady-state conductance of the sensor as a function of the H_2_ concentration

During exposure to increasing H_2_ concentrations ranging from 2 to 1000 ppm under the optimal operating temperature of 240 °C, the steady-state resistance response of the sensor is also investigated and shown in Fig. 6a. According to the Zhu et al. [16], this should be done in a stable environment, and the time of measurement should be longer enough to insure adsorption of hydrogen molecules to reach a steady state. Figure 6b shows that the corresponding steady-state conductance (*G* = 1/*R*) of the sensor follows a power law depending on the H_2_ gas concentration, *G*_gas_ = *G*_air_ + *α*(concentration)^*β*^, where *α* is a constant, *β* is a power exponent, and the ideal value is 0.5 [17]. The experimental data deduced from the measurement results in Fig. 6a is well consistent with the exponent fitting results, and the corresponding values of *α*, *β*, and correlation coefficient *R* obtained in the process of fitting are indicated in Fig. 6b. For the *β* value of 0.48578, the small deviations from the ideal value of 0.5 probably relate to either agglomeration or zones in the structure that are less sensitive to H_2_ than others, as modeled by Scott et al. [18].

**Fig. 7:**
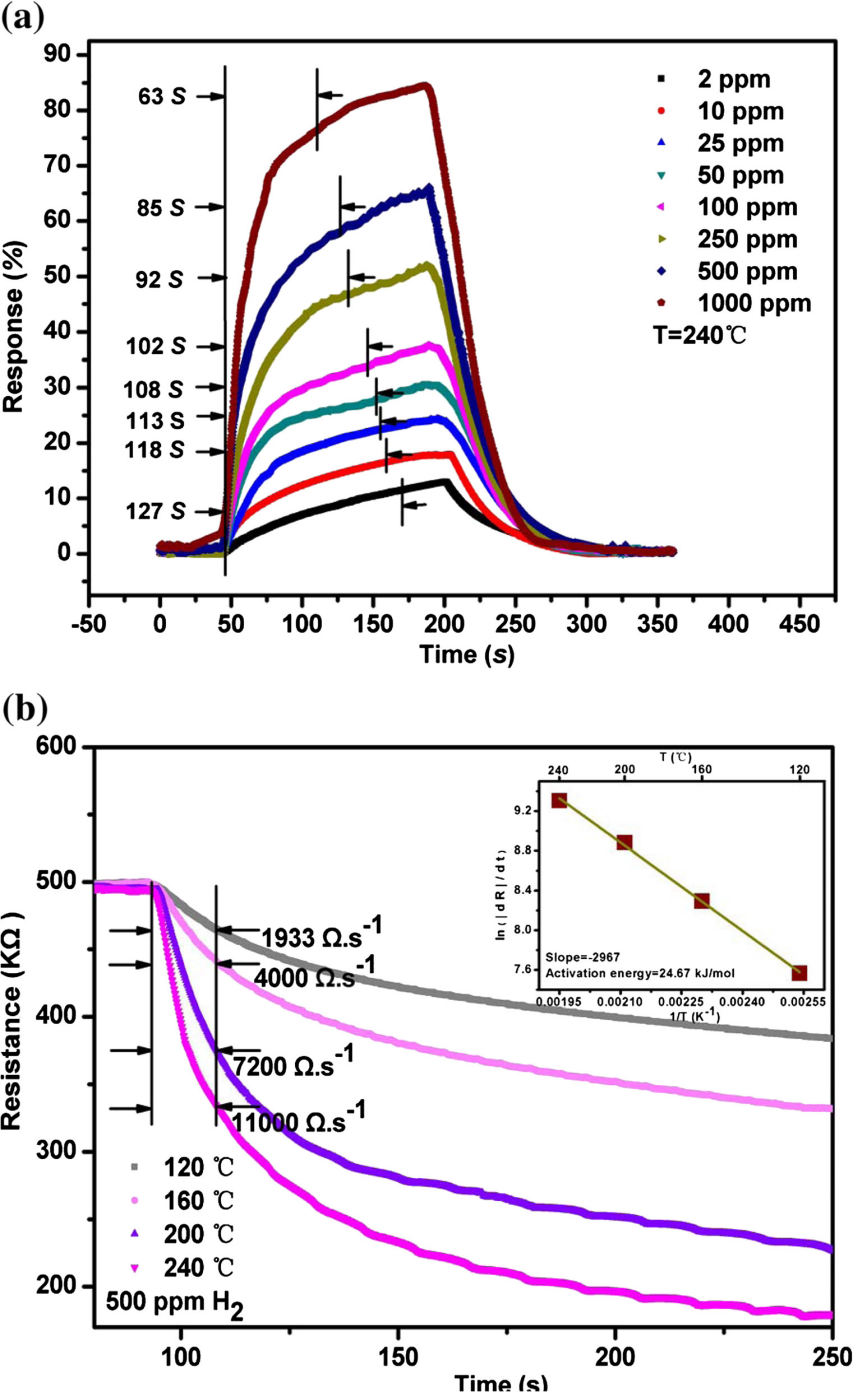
**a** One cycle of H_2_ in and out in the concentration range of 2–1000 ppm under an optimal operating temperature of 240 °C for the sensor. **b** Resistance vs. time on exposure to 500 ppm of H_2_ under different operating temperatures of 120–240 °C from the sensor, the *inset figures* showing the corresponding Arrhenius plot based on the rates of change of the resistance

Response time is also an important parameter for a gas sensor, which is defined here as the time reaching 90 % of the final equilibrium value [19]. The corresponding response time of the sensor exposed to H_2_ concentrations of 2–1000 ppm at the operating temperature of 240 °C is indicated in Fig. 7a. With the hydrogen concentration increasing from 2 to 1000 ppm, the response time decreases from 127 to 63 s. To be sure, although interdigitating gap is smaller, the response time is relatively longer compared with that of the relevant report [20]. As for the reasons, we would like to elaborate on two aspects. Firstly, it is related with the longer path between the gas mixer instrument and the test gas chamber as shown in Fig. 1h. Secondly, the relatively larger size of our nanotowers compared with that of relevant report [20] results in a correspondingly less surface-to-volume ratio, which can increase the response time too [20–22]. The time dependence of resistance of the In_2_O_3_ nanotowers on exposure to 500 ppm H_2_ at different operating temperatures of 120–240 °C is shown in Fig. 7b. The rate of resistance change becomes greater as the temperature increases. The inset figure shows the rate of Arrhenius plot for the resistance change of nanostructures. An adsorption activation energy of 24.67 kJ/mol for the In_2_O_3_ nanotowers as shown in Fig. 7b is larger than that of 11.8 kJ/mol for the Pd-coated ZnO nanowires [23], that of 2.2 kcal/mol for the Pd-coated GaN nanowires [24], and that of 23.6 kJ/mol for the Pt-coated W_18_O_49_ nanowire networks [16].

**Fig. 8:**
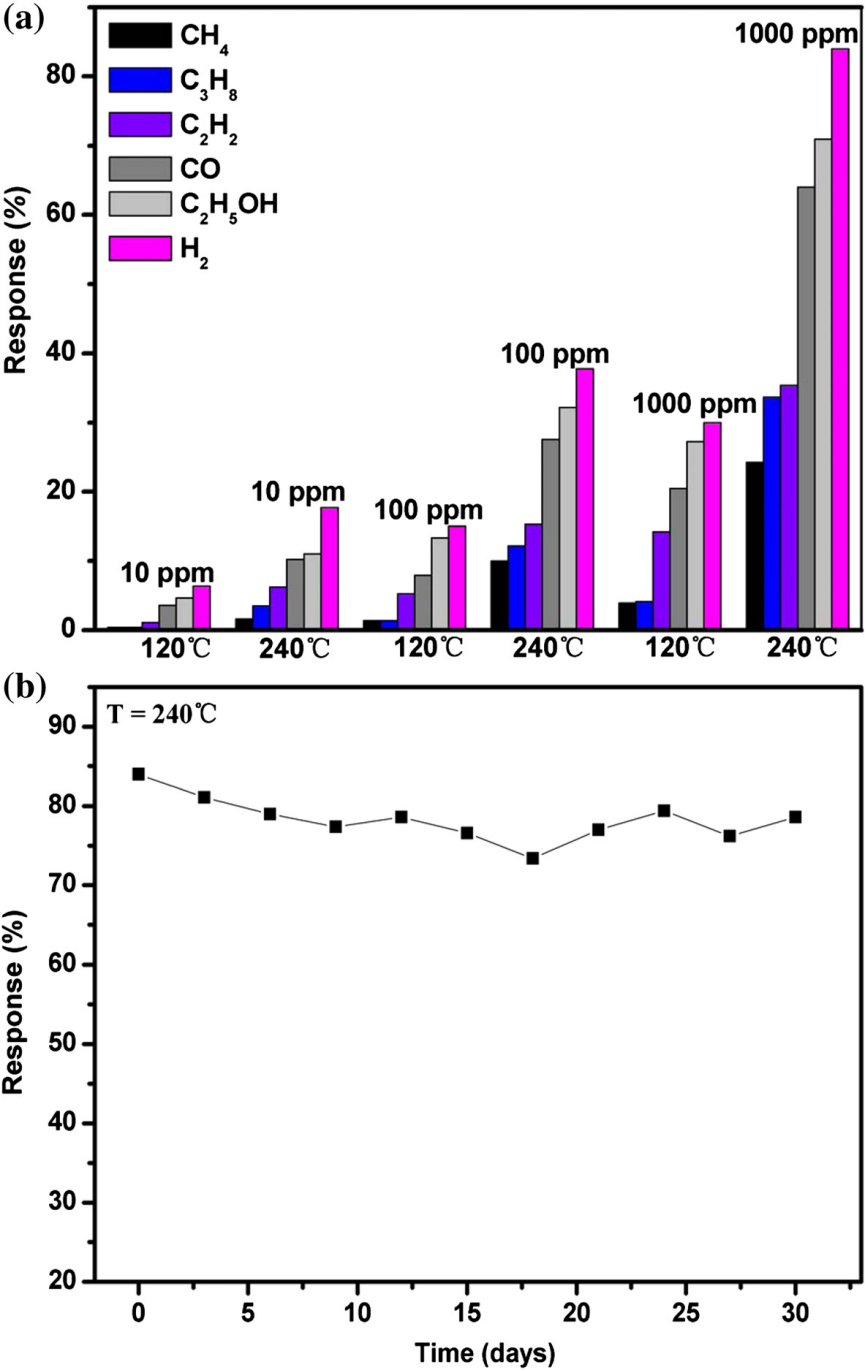
**a** The selectivity properties of the sensor to reducing gases such as CH_4_, C_3_H_8_, C_2_H_2_, CO, C_2_H_5_OH, and H_2_, at the concentration of 10, 100, and 1000 ppm and under the different operating temperatures of 120 and 240 °C. **b** Long-term stability of the sensor to 1000 ppm H_2_ at the operating temperature of 240 °C in 30 days

Selectivity of the gas sensor is very important in the gas-sensing application. As we know, In_2_O_3_ is one kind of broad-spectrum sensitive material. The selective property is tested by exposing the sensor in reducing gases such as CH_4_, C_3_H_8_, C_2_H_2_, CO, C_2_H_5_OH, and H_2_, respectively, which is shown in Fig. 8a. The concentration values are 10, 100, and 1000 ppm, and the operating temperatures are 120 and 240 °C. It is very clear to discover that the sensor exhibits much higher responses to H_2_ in comparison to that of CH_4_, C_3_H_8_, and C_2_H_2_, respectively, which is due to the reason that the hydrogen with the smallest molecular size can easily pass through the surface dense layer and react intensely with the negatively charged oxygen adsorbates on the In_2_O_3_ surface [20]. However, the sensor exhibits slightly higher responses to H_2_ than that of CO and C_2_H_5_OH, respectively, which is largely related with the activation energy of CO and C_2_H_5_OH being close to hydrogen. So, further study should be focused on improving the selectivity of the In_2_O_3_ nanotower gas sensors through surface modification with noble metals (Pt, Pd, Au, etc.). In addition, temperature modulation is also another efficient method for improving the selectivity of the metal oxide gas sensors [25]. Moreover, in order to improve the selectivity of the sensor to the macromolecular gas such as CO or C_2_H_5_OH, we take an effective method to install the activated carbon or zeolites on the cover of the sensors.

The long-term stability of the sensor is also displayed in Fig. 8b. We measure the response of the sensor to 1000 ppm H_2_ at an operating temperature of 240 °C every 3 days. Clearly, the sensor shows relatively stable response in 30 days.

Hydrogen-sensing response of the In_2_O_3_ nanotower gas sensor operated at 240 °C in comparison to those of the reported sensors using In_2_O_3_-based nano- or micro-structured materials is shown in Table 1. The range of the Response% of all the sensors normalized to (*R*_o_ − *R*_g_)/*R*_o_*100 % is from 0 to 100 % due to *R*_g_ smaller than *R*_o_ and greater than 0. Thus, these results definitely show that the In_2_O_3_ nanotower hydrogen sensor has basically achieved better responses than those of the reported sensors [3, 26–38].

**Table 1:** Hydrogen-sensing response of the In_2_O_3_ nanotowers sensor operated at 240 °C in comparison to those of the reported sensors using In_2_O_3_-based nano- or micro-structured materials

Type	Operated temperature (°C)	Response% (Δ*R*/*R*_o_*100 %) toward H_2_ under the following concentrations (ppm)						
		2	10–50	150–270	400–500	1000	2000	4000
In_2_O_3_ nanotowers	240	13	21	50	64	83		
In_2_O_3_ nanowires [3]	400			17	60			
In_2_O_3_ nanoneedles [3]	350			10.7	20			
ZnO/In_2_O_3_ nanorods [26]	20–25		9.8	15.5	20.5			
In_2_O_3_ thin films [27]	50				6.5			
In_2_O_3_ nanowires [28]	200				10			
Au-loaded In_2_O_3_ nanofibers [29]	140				33			
In_2_O_3_ hollow nanospheres [30]	300			41				
In_2_O_3_ nanowires [31]	300						69	
In_2_O_3_/ZnO nanowires [32]	400						83	
In_2_O_3_ nanowires [32]	300							71
In_2_O_3_ flower-like microspheres [33]	150					10		
In_2_O_3_ urchin-like microspheres [34]	150					10		
In_2_O_3_/La/Pd [35]	100				3			
In_2_O_3_ nanofibers [36]	300			43				
In_2_O_3_ nanowires [37]	250					17.9		
In_2_O_3_:Se [38]	–					40		

**Fig. 9:**
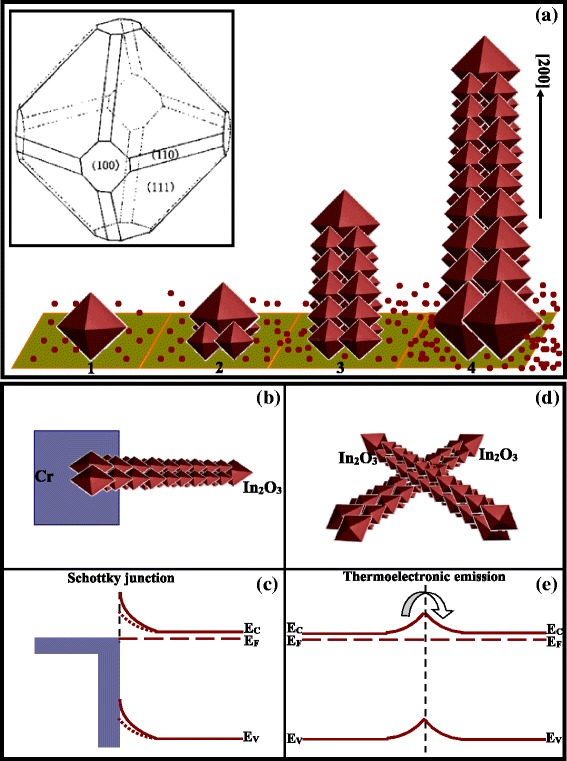
**a** The growth mechanism of the In_2_O_3_ nanotower, the *inset figure* is the schematic illustration of 0-D growth with octahedral configuration under the VS mechanism [39]. **b** The Cr/In_2_O_3_ Schottky junction and **c** the corresponding energy-level diagram. **d** The contact between two In_2_O_3_ nanotowers and **e** the corresponding energy-level diagram

The growth mechanism of the In_2_O_3_ nanotowers can be described to be the VS process, and the corresponding schematic illustration is shown in Fig. 9a. It is well-known that the growth rate perpendicular to different planes is proportional to their surface energies. For In_2_O_3_ with a bcc structure, the surface energy relationships among three low-index crystallographic planes should correspond to *γ*{111} < *γ*{100} < *γ*{110} [39]. The shape of the pyramid tip is consistent with the cubic crystal structure of In_2_O_3_ [40]. In the In_2_O_3_ cubic structure, the {001} family planes contain the three equivalent planes (100), (010), and (001), which are perpendicular to the three directions [100], [010], and [001], respectively. Pyramids bounded by {111} facets with the highest (111) to that of (100) surface energy ratio possess the lowest surface energy [40]. The difference of surface energies among {110}, {100}, and {111} facets can lead to their different growth rates, and the growth rates of three growth directions have such relationships: *r*_[111]_ < *r*_[100]_ < *r*_[110]_ [39]. As shown in the inset figure in Fig. 9a, after the 0-D growth, the (110) plane will disappear earlier, and the (111) plane will be preserved [39]. At the initial stage of heating, the indium vapor gradually evaporates out of the In oxide through a carbothermal reduction reaction, so the saturation ratio is low at this stage. Under a low saturation ratio, the 0-D nucleation and growth begin, and an octahedron forms under a VS mechanism [39], which is shown in the first process. According to a self-catalyzed effect, the surface polarity of the (111) plane of the cubic lattice in In_2_O_3_ octahedron can depress the relatively high nucleation potential barrier of In_2_O_3_ at the low temperature and induce the In_2_O_3_ nucleation on the plane [41]. Therefore, the four little 0-D In_2_O_3_ octahedrons can be formed at the four (111) plane of the big In_2_O_3_ octahedron, which is shown in the second process. During the 0-D growth, the indium vapor is uninterruptedly evaporated, so the saturation ratio gradually increases and finally reaches the critical value of the 1-D growth. Then, the 1-D growth at the top plane of the four little 0-D In_2_O_3_ octahedrons along the [200] direction begins. The 1-D growth consumes abundant reagent species and results in a decrease in the saturation ratio, so the 1-D growth stops and new 0-D growth begins. The 0-D growth will make the newly formed four little octahedral configurations. During the 0-D growth, the saturation ratio increases, and a new round of 1-D growth begins [39]. After the alternating 0-D and 1-D growths, the body of tower-shaped nanostructure continuously grows out, which is shown in the third process. As the reaction between the In_2_O_3_ powders and the active carbon from the initial stage develops, the reagent’s vapor pressure turns bigger and bigger. The continuously increasing reagent’s vapor pressure will continuously lead to large lateral and small axial growth rates, which will induce to the formation of 1-D structure with continuously increased size [42]. Therefore, the size of the In_2_O_3_ nanotowers can be continuously increased from top to bottom as shown in the fourth process.

The unique hydrogen-sensing properties based on the In_2_O_3_ nanotower can be explained as follows. In the open air, the chemisorbed oxygen ions (O_2_^−^ or O^−^) form on the surface of the In_2_O_3_ nanotowers leading to a depletion region in surface layer owing to the electron shift from In_2_O_3_ to oxygen. When the hydrogen is injected, H_2_ reacts with chemisorbed oxygen ions, which releases electrons back to the surface of the In_2_O_3_ nanotowers and alter its electrical conductivity of the structures. According to the XPS spectrum of O1s shown in Fig. 3c, it appears that a high concentration of chemisorbed oxygen ions will favor the forward reaction [2]. For example, it is found that the ethanol-sensing responses of In_2_O_3_ increase with the increasing intensity of chemisorbed oxygen [9]. In addition, because of the response of the metal oxide sensor to methanol depending on the basicity of the metal oxide [43] and the basicity controlled by the electronic density which is generated from the V_o_ [9], the hydrogen-sensing properties of the In_2_O_3_ nanotowers are excellent due to lots of V_o_ being introduced in the In_2_O_3_ nanotowers in this paper.

The hydrogen-sensing mechanism of the sensors is attributed to not only the hydrogen-sensing properties based on the In_2_O_3_ nanotower discussed above but also the structure characteristics of the sensors as schematically shown in Fig. 9b–e. Besides the change of the Schottky barrier height in the Cr/In_2_O_3_ Schottky junction as shown in Fig. 9b, c, the thermoelectronic emission due to the contact between two In_2_O_3_ nanotowers as shown in Fig. 9d, e will contribute for the hydrogen-sensing mechanism too.

The electrical response comes from the variation of the Schottky barrier height and barrier width as a result of adsorption of gaseous species at the Schottky contact. The response due to adsorption can be explained from the band diagram at the metal/nanostructure contact. After the exposure to H_2_, Cr adsorbs H_2_ by catalytic chemical adsorption, which reduces the Schottky barrier height [14]. The catalytic effect of chromium can be understood in two ways [44]. The first one is related to the work function value of the chromium which leads to an excess of free bonds near the surface region, then producing an important oxidation which favors the subsequent reactions with the H_2_. The second one is related to the formation of a Cr_2_O_3_ phase close to the surface region, which increases the catalytic activity of this region due to the fact that the surface chromium atoms can be easily oxidized and reach an effective valence of four. So, they can adsorb a complete monolayer of active oxygen.

In an individual In_2_O_3_ nanotower, free carriers (electrons) can transport along the conduction channel. But when transporting between two contacting In_2_O_3_ nanotowers, the electrons have to pass through a potential barrier at the junctions and a thermoelectronic emission mechanism can be used to describe the electron transportation in the junctions [16]. The change in resistance during the adsorption or desorption process of H_2_ species is likely to be caused by the alteration both in the width of the surface depletion layer of each In_2_O_3_ nanotower and in the height of potential barriers built at the contacted junctions between two In_2_O_3_ nanotowers. These two-fold effects may facilitate less response time and higher response to certain chemical species [45].

## Conclusions

In summary, we report that new In_2_O_3_ nanotowers synthesized via thermal evaporation and distributed on Cr comb-shaped interdigitating electrodes with relatively narrower interspace of 1.5 μm have high response, fine long-term stability, and small deviation from ideal value of power exponent *β*, in the hydrogen concentration ranging from 2 to 1000 ppm and at the operating temperature of 120–275 °C. The Schottky contact between the In_2_O_3_ nanotower and the Cr comb-shaped interdigitating electrode forms the Cr/In_2_O_3_ nanotower Schottky diode, and the corresponding temperature-dependent *I*-*V* characteristics have been measured. The diode exhibits a low Schottky barrier height of 0.45 eV and ideality factor of 2.93 at room temperature. The growth mechanism of the In_2_O_3_ nanotowers has been discussed in detail, which is account for the high surface-to-volume ratio of the morphology. Lots of oxygen vacancies and chemisorbed oxygen ions exist in the In_2_O_3_ nanotowers according to the XPS results. The change of Schottky barrier height in the Cr/In_2_O_3_ Schottky junction and the thermoelectronic emission due to the contact between two In_2_O_3_ nanotowers mainly contribute for the H_2_ sensing mechanism. The In_2_O_3_ nanotowers hydrogen sensors are promising for further practical applications.
